# High anticancer efficacy of solid lipid nanoparticles containing *Zataria multiflora* essential oil against breast cancer and melanoma cell lines

**DOI:** 10.1186/s40360-021-00523-9

**Published:** 2021-09-30

**Authors:** Alireza Valizadeh, Ali Asghar Khaleghi, Ghazaal Roozitalab, Mahmoud Osanloo

**Affiliations:** 1 Department of Medical Nanotechnology, School of Advanced Technologies in Medicine, Tehran University of Medical Sciences, Tehran, Iran; 2 Noncommunicable Diseases Research Center, Fasa University of Medical Sciences, Fasa, Iran; 3 Department of Medical Nanotechnology, School of Advanced Technologies in Medicine, Fasa University of Medical Sciences, Fasa, Iran

**Keywords:** Malignancy, Cell Survival, Phytochemicals, Herbal Medicine

## Abstract

**Background:**

The cancer burden is rising rapidly worldwide, and it annually causes about 8.8 million deaths worldwide. Due to chemical drugs’ side effects and the emergence of resistance, the development of new green drugs has received much attention. We aimed to investigate whether solid-lipid nanoparticles containing essential oil of *Zataria multiflora* (ZMSLN) enhanced the anticancer efficacy of the essential oil against breast cancer (MDA-MB-468) and melanoma (A-375) cells.

**Results:**

ZMSLN was prepared by the high-pressure homogenizer method; particle size 176 ± 8 nm, polydispersity index 0.22 ± 0.1, entrapment efficiency 67 ± 5%. The essential oil showed a dose-dependent antiproliferative effect on MDA-MB-468 and A-375 cells at all examined concentrations (75, 150, 300, 600, and 1200 μg/mL). Interestingly, after treating both cells with 75 μg/mL of ZMSLN, their viabilities were reduced to under 13%.

**Conclusion:**

The finding showed that ZMSLN had a distinct antiproliferative efficacy; it could thus be considered a green anticancer candidate for further in vivo and in vivo studies.

## Introduction

Cancers have many financial, emotional, and physical burdens in all societies and comprise approximately 17% of global deaths [1]. Although cancer incidence trends are declining by 3.1%/year in men, it was stable in women (from 2009 to 2012) [2]. Cardiovascular disease and cancer are the first and second leading causes of death worldwide [3]. Breast cancer is defined as uncontrolled cell proliferation or growth, becoming the most prevalent cancer in many Asian countries [4, 5]. Melanoma, or cancer of melanocytes in skin tissue, is another prevalent form of cancer with an increasing rate in the last decades worldwide [6]. Due to its heavy burdens on societies in different countries, more attention is required to prevent melanoma morbidity, incidence, and mortality [7].

Bone marrow depression, extreme fatigue, alopecia, loss of self-esteem or immunity, and a remarkable decline in white blood cell count are common side effects of synthetic or semisynthetic anticancer drugs [7, 8]. Thus, the identification and development of novel anticancer using natural products with fewer serious adverse effects have received more attention [9, 10]. *Zataria multiflora* Bioss, the *Lamiaceae* family, is one of the most important medicinal plants in Pakistan, Afghanistan, and southern Iran; it is applied as an antiseptic and antitussive in respiratory tract disorders [11]. The *Z. multiflora* essential oil (ZMEO) also possesses many biological activities, including anticancer and antioxidant effects [11, 12]. EOs (such as ZMEO) are generally hydrophobic; loading them into nano-carriers is one of the most effective ways to improve their performance in laboratory and animal researches [13, 14]. Solid-lipid nanoparticles (SLN) possess advantages over other colloidal systems, such as improved physical stability and sustaining the drug release [15, 16]. We describe here SLNs containing ZMEO (ZMSLN) model for natural indexing products with anticancer effects against breast cancer (MDA-MB-468) and melanoma (A-375) cells.

## Materials and Methods

MDA-MB-468 (ATCC HTB-132 and A-375 (ATCC CRL-1619) as breast cancer and melanoma cell lines were purchased from Pasteur Institute of Iran. Stearic acid, tween 80, span 60, phosphate-buffered saline (PBS) tablets, penicillin-streptomycin, trypsin, dimethyl sulfoxide (DMSO), absolute ethanol (99.8%), and MTT (3- (4, 5-dimethyl thiazolyl-2)-2, 5diphenyltetrazolium bromide) were bought from Merck Co. (Germany). Shellmax (China) and Gibco (USA) supplied cell culture medium (RPMI 1640) and fetal bovine serum (FBS). ZMEO was bought from Zardband Pharmaceutical Co. (Iran).

### Preparation of ZMSLN

ZMSLN was prepared according to our described high-pressure Homogenizer method [17]. ZMEO (1% v/v) was first dissolved in stearic acid 4% w/v (temperature = 85 °C) and a hot span 60 2% w/v (lipophilic surfactant). After that, the prepared mixture was dispersed in a hot surfactant solution (tween 80—4%) and homogenized (1 min, 8000 RPM) using a high-shear Homogenizer (D-91126 Schwabach, Heidolph, Germany). The obtained pre-emulsion was then homogenized, three cycles, at high pressure, 500 bar, using an APV Micron Lab 40 (APV Systems, Unna, Germany) thermostated at 90 °C. Moreover, free SLNs were also prepared in the same procedure; only ZMEO was not used.

### Investigation particle size and morphology of the prepared SLNs

The size of ZMSLN and free SLNs were analyzed by Dynamic Light Scattering (DLS, the model of 9900, K-one Nano Ltd., Korea). The formulation was poured into a quartz cell and situated in the hole of the device for analysis.

To determine the shape of ZMSLN and free SLNs, it was first diluted two times with distilled water; one drop was then impregnated on a copper grid (200-mesh carbon-coated). Next, it was stained with phosphotungstic acid (2%) solution, dried at room temperature, and subjected to a TEM device, Philips CM30 TEM microscope (Netherlands).

### Entrapment efficiency

ZMEO was first diluted in ethanol in a 150–350 μg/mL concentration, and absorbance was then screened (200–400 nm) to determine the maximum absorption wavelength (λ max). The standard calibration curve (with regression equation) for ZMEO was then plotted (see Fig. 3). For determining entrapment efficiency, ZMSLN was first centrifuged for 20 min at 25,000 RPM and then filtered using a syringe filter (pore size 0.22 μm; *n =* 3). ZMEO content in the supernatant was then determined by the UV-Visible method; absorbance at λ max was placed in the regression equation.

1 $$ Entrapment\ efficiency=\left( initial\ drug\ amount- drug\ supernatant/ initial\ drug\ amount\right)\times 100 $$

### MTT assay

The in vitro cytotoxicity of ZMEO and ZMSLN on the breast cancer (MDA-MB-468) and melanoma (A-375) cancer cells was estimated by MTT assay according to our previous study with slight modification [18]. ZMEO was dissolved (4800 μg/mL) in PBS solution containing 0.5% DMSO; required serial dilutions were also prepared using the same solvent. The cells were first seeded (1 × 10^4^ cells/well) in 96-well culture plates overnight. Wells content was then replaced with 75 μg.mL^−1^ RPMI complete medium (containing 10% FBS and penciling/streptomycin 1%). The cells were treated with various concentrations of ZMEO and ZMSLN (75, 150, 300, 600, and 1200 μg.mL^−1^), as well as free SLNs; treated plates were incubated for 24 h (37 °C and 5% of CO_2_ concentration). Wells’ content was then discarded and washed with PBS to the removed milky color of ZMSLN. After that, 100 μL of MTT reagent (0.5 mg/mL dissolved in RPMI) was treated for each well and incubated for 4 h at 37 °C and 5% of CO_2_ concentration. Finally, 100 μL/well DMSO was added to solubilization the precipitate formazan crystals to the obtained purple color. The absorbance (A) of wells was measured at 570 nm by the ELISA Plate Reader. Cell viabilities at different concentrations were calculated by eq. 2. Six well/plates were considered the control group, contained with PBS solution containing 0.5% DMSO (25 μL) and RPMI (75 μL).

2 $$ \mathrm{Cell}\ \mathrm{viability}=\left(A\  sample/A\  control\right)\times 100 $$

### Statistical Analysis

All the tests were carried out in triplicate; results were expressed as mean ± SD. CalcuSyn software (Free version, BIOSOFT, UK) was applied for calculating the IC_50_s of the samples. One-way ANOVA (SPSS software v. 22, USA) with the least significant difference test (L.S.D) at the 5% level was used to compare anticancer activities.

## Results

### Characterization of size, morphology, and entrapment efficiency

**Fig. 1:**
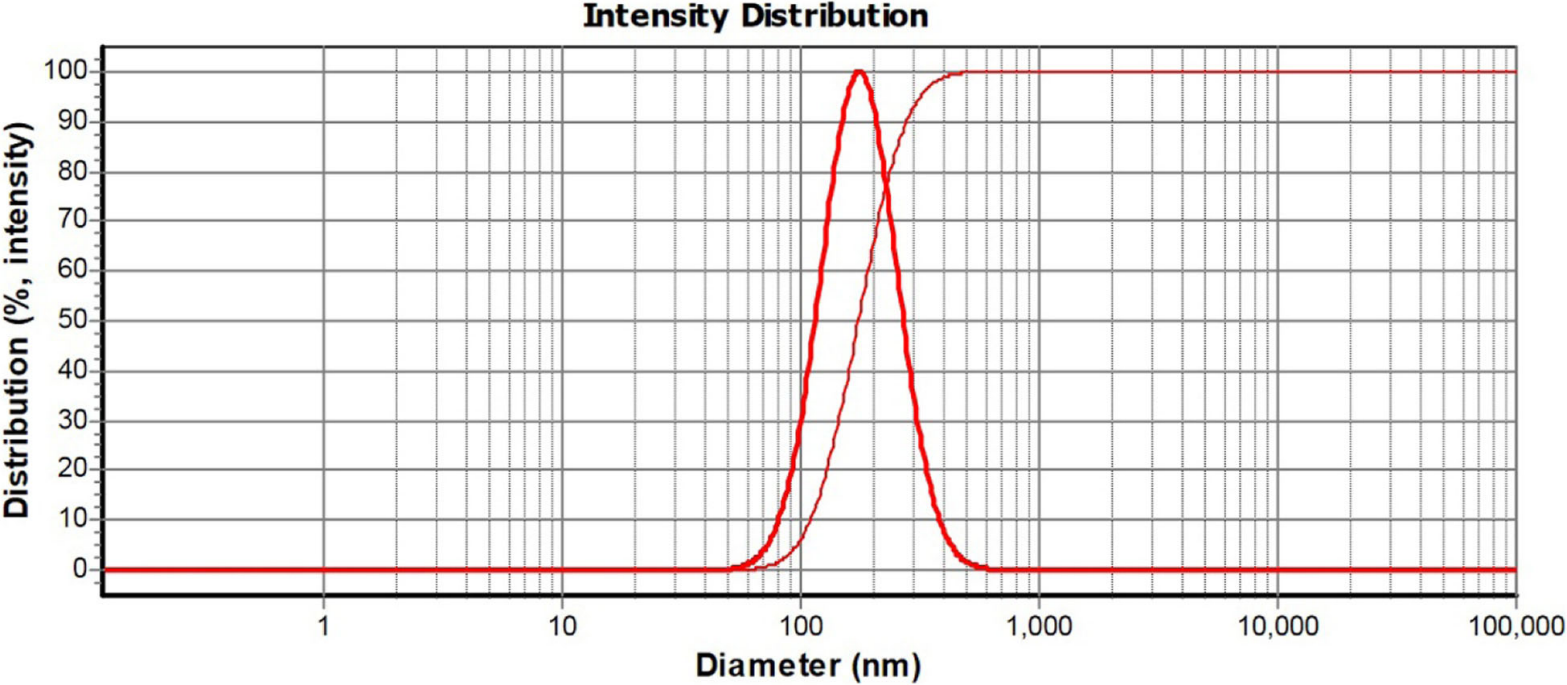
The DLS graph of solid-lipid nanoparticles containing *Z. multiflora* EO (ZMSLN)

**Fig. 2:**
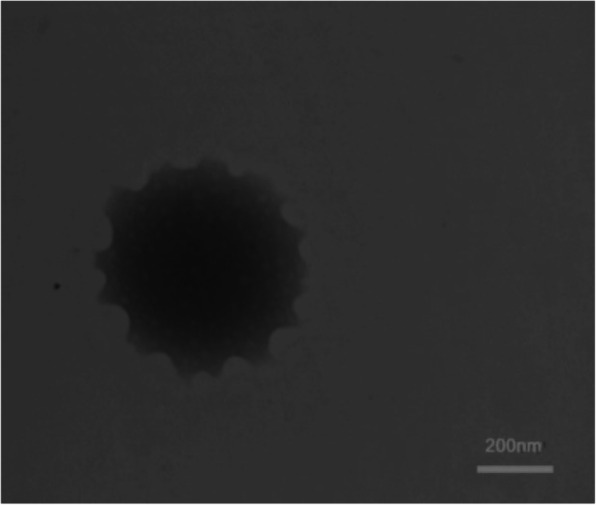
Image of solid-lipid nanoparticles containing *Z. multiflora* EO (ZMSLN). Scale bar = 200 nm

**Fig. 3:**
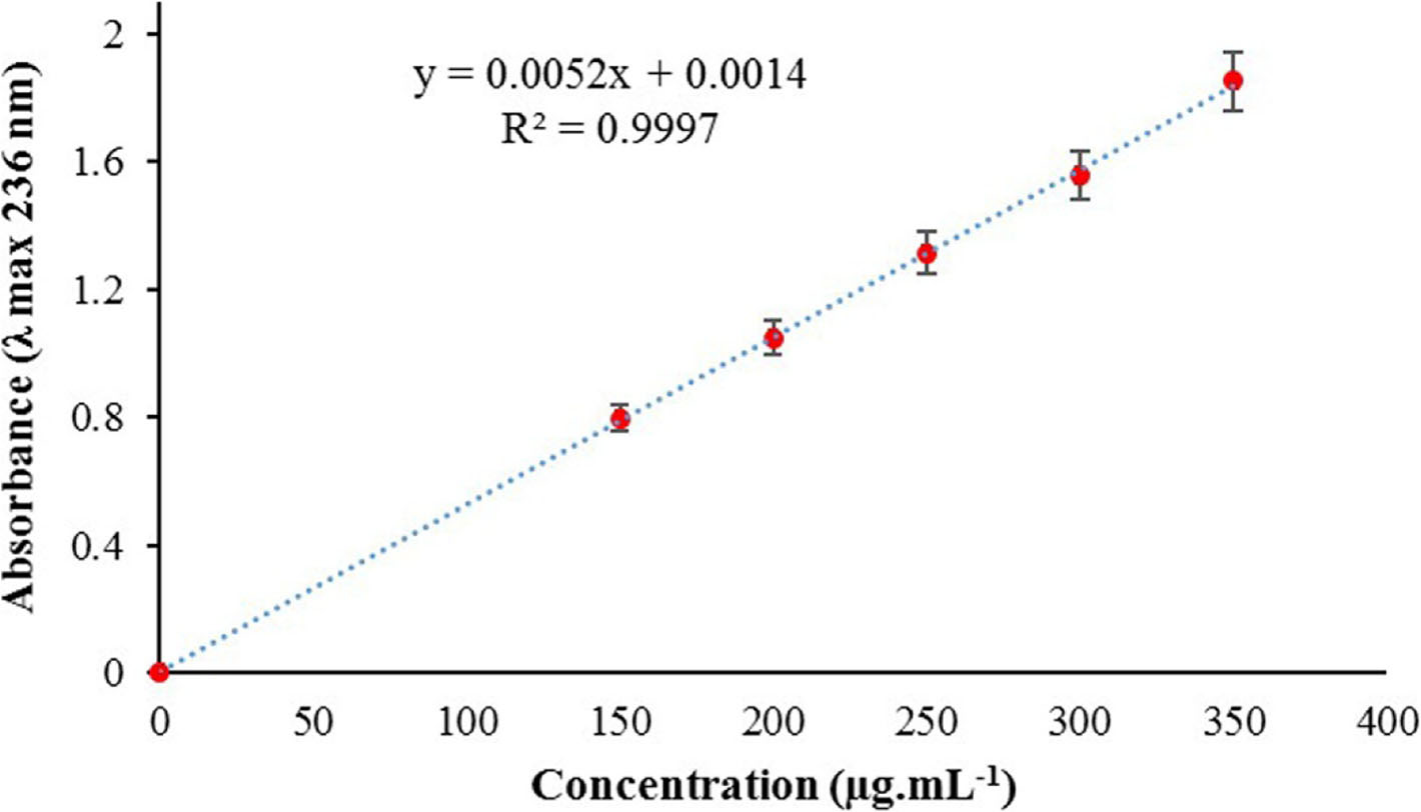
Calibration curve for determining the amount of *Z. multiflora* EO

DLS characterized the ZMSLN with a particle size of 176 ± 8 nm and a polydispersity index of 0.22 ± 0.1 (see Fig. 1). The morphology of ZMSLN was demonstrated by TEM; it showed a spherical shape with a toothed shell-like (see Fig. 2). Moreover, a polluted calibration curve for determining the amount of ZMEO is depicted in Fig. 3. Noted, λ max of ZMEO was obtained at 236 nm, and the entrapment efficiency was achieved at 67 ± 5%.

### In vitro anticancer activity

**Fig. 4:**
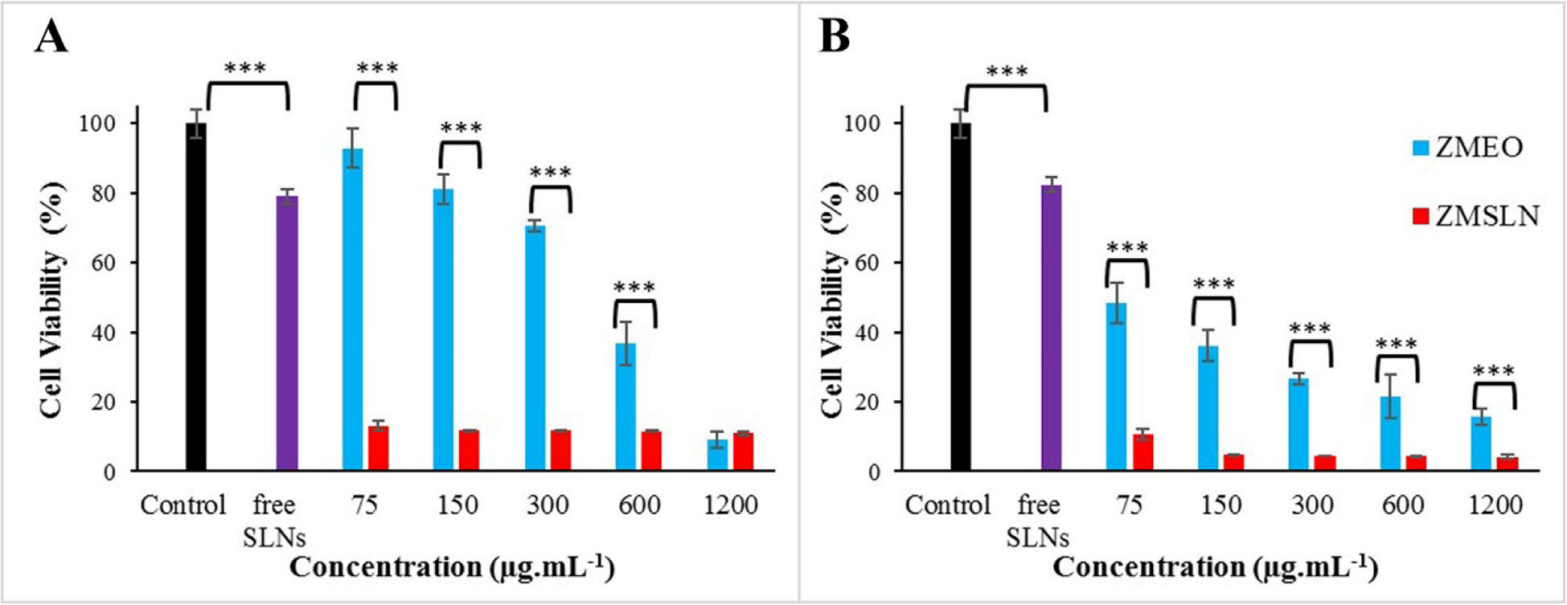
Viability of MDA-MB-468 (A) and A-375 (B) cells after treatment with solid-lipid nanoparticle without EO (free SLNs), *Z. multiflora* EO (ZMEO), and solid-lipid nanoparticle containing the EO (ZMSLN). ***Significant difference (*P* < 0.001)

As shown in Fig. 4A, MDA-MB-468 cancer cells have susceptibility to ZMSLN at all concentrations. Besides, the results showed that the survival of MDA-MB-468 cancer cells was under 50% at a concentration of ≥600 μg.mL^−1^ in the treating group with ZMEO. The difference between the anticancer activity of ZMEO and ZMSLN was significant (*P* < 0.001) at all concentrations. Moreover, a significant difference (*P* < 0.001) is observed amongst the control group and treated cells with free SLNs; viability was reduced to 79%.

From Fig. 4B, the viability of the A-375 cell line in the presence of ZMEO and ZMSLN showed a significant reduction under 50% at all concentrations. The results demonstrated that ZMSLN had significantly higher anticancer activity than ZMEO at all concentrations (*P* < 0.001). Besides, the viability of the cell after treatment with free SLNs was significantly (*P* < 0.001) reduced (to ~82%) in comparison with the control group.

IC_50_ values of ZMEO against MDA-MB-468 and A-375 cells were obtained at 380 and 59 μg.mL-1. Because even the lowest concentration of the ZMSLN reduced cell viability of both cell lines below ~ %13, IC_50_ of ZMSLN was thus not calculating precisely. However, it could be concluded that ZMSLN had a distinct antiproliferative effect.

## Discussions

Breast and skin cancer are the two most frequent types of cancers worldwide [19, 20]. The commonest life-threatening malignancy in women is breast cancer; it is the cause of 14% of all cancer-related fatalities [21, 22]. MDA-MB-468 without estrogen and progesterone receptors and HER_2_ non-expression (triple-negative) is an invasive and poorly differentiated cell line. It is one of the commonly used cell lines in the literature [23, 24]. Skin cancer is the most common and preventable carcinoma around the world, and rising annual incidence has made it a pre-eminent public health threat [25]. Malignant melanoma is a type of skin cancer and is responsible for most skin cancer deaths; it begins with the abnormal proliferation of cells known as melanocytes [26]. Amongst melanoma cell lines, A-375 cells are aggressive with low susceptibility to chemotherapy [27, 28].

Natural products, especially EOs, have been widely used for their therapeutic properties as chemopreventive/chemotherapeutic agents in vitro and in vivo studies [29–31]. For instance, in our previous study, chemical compositions of ZMEO (the used batch in the current study) was first investigated; carvacrol (30.2%), thymol (25.2%), o-cymene (10.7%), c-terpinene (6.1%), and α-pinene (3.616%) were identified as five major compounds. Its anticancer effects were then evaluated against breast cancer cell lines, including MCF-7, MDA-MB-175, and MDA-MB-231; IC_50_ values were obtained as 76, 70, and 104 μg.mL^−1^ [32]. Furthermore, the anticancer effect of major ingredients of some EOs has been repeatedly investigated on different cancer cells [33]. For instance, anticancer effects of ZMEO main constituents, i.e., thymol and carvacrol (monoterpenoids and monoterpenes), were reported [33–36]. For instance, thymol and carvacrol at lower concentrations had antioxidant activity and at higher doses had antiproliferative and apoptotic effects [37]. The cytotoxicity of carvacrol with IC_50_ 380 μM was more potent than thymol with IC_50_ 497 μM against human non-small cell lung carcinoma after a 24 h incubation period [37]. Recently anticancer effects of thymol and carvacrol were investigated against the SKOV-3 ovarian cancer cell line by *Elbe et* al.; thymol was more potent than carvacrol [38].

SLNs contain a hydrophobic solid matrix core with one layer of a phospholipid coating [39]. The high entrapment efficiency for hydrophobic drugs in the core, well-tolerated composition due to physiologically similar lipids, prolonged drug release feature, low toxicity, biodegradable composition, and high stability makes SLNs promising candidates for cancer therapy and pharmaceuticals usage [40, 41]. It was thus chosen in the current study to entrap ZMEO inside the core. However, some reports on the loading of EOs into SLNs have been found. In one research, SLNs containing *Croton argyrophyllus* EO with the particle size of 201.4 ± 2.3 nm and entrapment efficacy 89.63 ± 1.02% was reported; an improvement in antioxidant properties of EO in the treatment of neurodegenerative diseases was observed [42]. In another research, SLNs containing ZMEO were prepared to increase its antifungal activity efficiency. The entrapment efficacy of ZMSLN was 64.6 ± 3.8%, with a particle size of 134 ± 7 nm. ZMSLN also facilitated the applicability of ZMEO as antimicrobials and antifungal medication [43, 44]. SLNs loaded with *Eugenia caryophyllata* EO were presented a unique nanoparticulate system with a vast antimicrobial activity. The particle size was between 397 ± 10 nm and 786 ± 11, with entrapment efficacy at approximately 70% [43]. Another study aimed to prepare SLNs (with a mean size of 113.3 ± 3.6 nm and entrapment efficacy of 80% ± 1%) for the oral delivery of two EOs. The SLNs increased the antitumoral activity of EOs in H22-bearing Kunming mice [44]. In 2013, Moghimpour *et al.* designed and prepared a SLN from ZMEO using hot homogenization and precipitation methods; the prepared SLNs had a mean size of 650 nm [45]. Ehsanfar *et al.* applied the ultrasonic Homogenizer method to produce several SLNs with different sizes [46].

Furthermore, several studies showed that formulating EO in nanocarriers leads to higher activity. For example, Nasseri *et al.* prepared ZMSLN with a particle size of 255 ± 3 nm to improve and control antifungal efficiency in some fungal pathogens [47]. In our previous study, antibacterial activity increased by reducing the particle size of two EOs of Zataria multiflora and Mentha piperita [48]. In addition, the leishmanicidal activity of *Cinnamomum zeylanicum* EO against *Leishmania tropica* and *Leishmania major* was increased when size was reduced to the nanoscale range, 52 ± 4 nm [18]. Also, curcumin-loaded SLN had greater antiproliferative activity on breast cancer cells than the bulk of curcumin [49]. Besides, the anticancer activity of variabilin was improved by reducing the size to nano range (IC_50_ 8.94 μM) compared with free variabilin (IC_50_ 87.74 μM) against the PC-3 cell line [50].

In the current study, the anticancer activity of ZMEO and ZMSLN was evaluated; results represented that ZMEO at the nanoscale size (176 nm) had a substantially more anticarcinogenic effect compared with bulk form against MDA-MB-468 and A-375 cancer cell lines. It may relate to the higher efficiency of cellular uptake and intracellular absorption of ZMSLN than the bulk form of ZMEO [51]. Moreover, in the current study, no dose-response effects were observed in cells treated with ZMSLN; viability of MDA-MB-468 cells was reduced to ~10%, and A-375 cells viability was reduced to ~5% at a concentration range of 150–1200 μg.mL^−1^. This is due to the maximum capacity of this nanoformulation at exanimated concentrations; with decreasing concentrations, lower efficacies become visible. In our previous report, the anticancer properties of chitosan nanoparticles containing *Citrus sinensis* and *Citrus limon* essential oils at exanimated concentrations were also not dose-dependent [52]. However, due to the promising efficacies of the prepared nanoformulation (ZMSLN) in a wide range of concentrations (150–1200 μg.mL^−1^), further research to investigate toxic effects on normal cells and other cancer cell lines in vitro and animal models are suggested.

## Conclusions

Solid-lipid nanoparticle-containing *Z. multiflora* EO (ZMSLN) with a particle size of 176 ± 8 nm, polydispersity index 0.22 ± 0.1, and entrapment efficiency 67 ± 5% was first prepared. The comparative study showed that ZMSLN had a higher anticancer activity on tested cancer lines (MDA-MB-468 and A-375) than the EO treatment group. Furthermore, after treating both cell lines with the lowest examined concentration of ZMSLN, 75 μg/mL, their viabilities were decreased under %13. ZMSLN could thus be introduced as a green potent antiproliferative candidate. However, more studies should be evaluated to reveal the exact mechanism of anticancer activity of ZMSLN.

## Data Availability

All data generated or analyzed during this study are included in this published article.
